# Legumes and nuts intake in relation to metabolic health status, serum brain derived neurotrophic factor and adropin levels in adults

**DOI:** 10.1038/s41598-023-43855-8

**Published:** 2023-09-30

**Authors:** Mohammad Javad Assi, Donya Poursalehi, Shahnaz Amani Tirani, Farnaz Shahdadian, Zahra Hajhashemy, Elahe Mokhtari, Sobhan Mohammadi, Parvane Saneei

**Affiliations:** 1 Students’ Research Committee, Isfahan University of Medical Sciences, Isfahan, Iran; 2 Department of Community Nutrition, School of Nutrition and Food Science, Nutrition and Food Security Research Center, Isfahan University of Medical Sciences, PO Box 81745-151, Isfahan, Iran; 3 Department of Clinical Nutrition, School of Nutrition and Food Science, Nutrition and Food Security Research Center, Isfahan University of Medical Sciences, Isfahan, Iran

**Keywords:** Nutrition, Endocrine system and metabolic diseases

## Abstract

There is controversial evidence about the relationship between consumption of legumes and nuts with metabolic disturbances. The present study was undertaken to explore the association of legumes and nuts intake with metabolic health status among Iranian adults. This cross-sectional study was conducted on 527 adults (45.7% female, aged 20–65 years) chosen through a multistage cluster random-sampling approach. Dietary intakes of individuals were assessed using a validated food frequency questionnaire. Fasting blood samples were gathered to evaluate biochemical parameters. Metabolic health status of subjects was determined according to the criteria defined by Wildman. Data of covariates were collected using pre-tested procedures. The overall prevalence of metabolic unhealthy (MU) phenotype was 42.5%. After controlling all confounders, participants with highest intake of legumes and nuts had lower odds of MU status, compared with the lowest intake (OR 0.35; 95% CI 0.18–0.71). This association was stronger in normal-weight rather than overweight/obese adults and also in women rather than men. Higher consumption of legumes and nuts was additionally related to decreased odds of hyperglycemia, hypertriglyceridemia, and hypertension. A marginally inverse association was observed between legumes and nuts intake with low brain-derived neurotrophic factor (BDNF) levels, in fully-adjusted model (OR_T3 vs. T1_ 0.50; 95% CI 0.25–1.01). Each tertile increase in legumes and nuts intake was marginally related to higher adropin levels ($\beta$ = 4.06; P = 0.07). In conclusion, this study demonstrated that higher intake of legumes and nuts is associated with lower chance of MU both in normal weight and overweight/obese adults. The association may be facilitated through serum BDNF and adropin.

## Introduction

Obesity, currently affecting about one-third of the world’s population, is considered as a major public health threat, due to its effect on risk of chronic diseases such as type 2 diabetes mellitus, cardiovascular diseases (CVDs), and some types of cancers^1–3^. However, recently the concept of metabolic health status has challenged the role of obesity in determining risk of chronic diseases. To be more exact, a subgroup of obese individuals with a favorable metabolic profile who are known as metabolically healthy obese (MHO) is less likely to have chronic diseases compared to metabolically unhealthy obese (MUO) individuals^4^. Additionally, a high risk of cardiometabolic disorders has been reported in normal-weight individuals with a metabolically unhealthy (MU) profile^5,6^. Therefore, it seems that metabolic status could be a better predictor for risk of chronic diseases than obesity, and maintaining a healthy metabolic status could be considered a beneficial strategy for reducing risk of chronic diseases.

During recent decades, emerging evidence has pointed to the metabolic role of hormones such as adropin and brain-derived neurotrophic factor (BDNF). Findings of experimental investigations indicated that these secreted molecules could control metabolism of glucose and lipid and regulate energy hemostasis^7,8^. Decreased serum levels of adropin and BDNF have been also reported in people with metabolic disorders such as obesity, diabetes mellitus, and CVDs^9–11^. Some reports have also indicated that modifiable factors such as dietary intakes and physical activity could affect adropin and BDNF levels^12–16^. It is postulated that adropin and BDNF play key roles in determining metabolic health status. Therefore, altering their serum levels is probably one of the mechanisms through which behavioral interventions improve metabolic health status.

Previous studies have suggested lifestyle modification as a beneficial approach for improving metabolic parameters. During recent decades, many epidemiological and clinical studies have highlighted the role of diet, among other lifestyle factors, on metabolic status^17^. A great body of evidence from observational and interventional studies has demonstrated the beneficial role of healthy dietary patterns, such as Mediterranean diet, dietary approach to stop hypertension (DASH) diet and plant-based diets on metabolic status^18–20^. These dietary patterns are rich in fruits, vegetables, whole grains, legumes, and nuts. Among plant-based foods, legumes, and nuts may affect the metabolic parameters potentially, due to their favorable content of nutrients and bioactive compounds such as vegetable protein, fiber, unsaturated fatty acids, vitamins, minerals, phytosterols, and phytochemicals^21,22^.

The relationship between dietary intake of legumes and nuts with metabolic syndrome (MetS) or its components has been investigated in previous studies. However, the results of these studies are contradictory. Some epidemiological evidences suggest that dietary intake of legumes and nuts is associated with a reduced risk of MetS or its components^23–27^, while other studies have shown conflicting results^28–32^. A recently published investigation has evaluated the link of nuts and legumes intake with metabolic health status in Iranian adolescents^33^, but no previous study has investigated this relationship among adults, especially in Middle Eastern populations. Therefore, the purpose of this study was to investigate the association of nuts and legumes intake with metabolic health status in Iranian men and women adults, considering the potential underlying role of BDNF and adropin.

## Materials and methods

### Study design and population

This cross-sectional study was performed in 2021, on a sample of Iranian adults living in Isfahan, Iran. According to a multistage cluster random-sampling approach, all adults working as teachers, school managers, employees, assistants or crews in several schools of different educational districts of Isfahan were selected. Based on a previous published study^34^, 49.4% of Iranian adults might have metabolic disorders; so, considering type 1 error of 0.05 (confidence interval (CI) of 0.95), precision (d) of 4.5% and power of 80%, 474 subjects were minimally required for this study. Nevertheless, due to high prevalence of covid-19 pandemic and its potential impact on data collection, 600 individuals were invited to participate in our investigation. Response rate was 90.5%. Individuals with the following characteristics were not included in our study: (1) having a history of type 1 diabetes, cardiovascular diseases, stroke and cancer; (2) being pregnant or lactating; (3) following a special diet. Exclusion criteria were: (1) having left more than 70 items on the food frequency questionnaire (FFQ) unfilled; (2) reporting energy intake outside of 800–4200 kcal/day; (3) refusing blood draw. Finally, a total of 527 adults aged 20 to 65 years old were eligible to be included in this analysis. Each participant signed a written informed permission. Local Ethics Committee of Isfahan University of Medical Sciences approved the protocol of this study (no. 3402104).

### Assessment of dietary intakes

We evaluated dietary intakes of participants using a Willett-format semi-quantitative 168-item FFQ. Validity of this questionnaire has been approved through a prior validation research which found reasonable correlations between the food intakes determined by this questionnaire and those acquired from several 24-h dietary recalls^35^. A comparison of nutrient intakes derived from this FFQ on two occasions, 1 year apart, indicated reliability of this tool as well^35^. All Participants were guided by a registered dietitian to fill out the FFQ by describing the frequency and amount of their eaten foods during the last year. Afterward, the portions of ingested items were changed to g/day using household measurements^36^. Lastly, data of all food items were input into the Nutritionist IV program to calculate daily energy and nutritional intakes.

### Assessment of anthropometric indices and blood pressure

Weight, height and waist circumference (WC) of participants were assessed by two skilled dietitians, with little clothing and no shoes. The body composition analyzer (Tanita MC-780MA, Tokyo, Japan) was utilized to determine weight (to the nearest 0.1 kg). Height was measured using a tape measure fixed on the wall (to the nearest 0.1 cm). Then, body mass index (BMI) was computed by dividing weight (kg) by height squared (m^2^). WC was evaluated after a normal breath with no outside pressure on the body and by applying an unstretched flexible tape measure (to the nearest 0.1 cm). Blood pressure (BP) of each person was taken after sitting for five minutes with an empty bladder and no prior exercise. A digital sphygmomanometer (OMRON, M3, HEM-7154-E, Japan) with an accuracy of 0.5 mmHg was used to assess BP. The measurements were conducted twice, with intervals of 5–10 min, and average of the measurements was reported as the final BP.

### Assessment of biochemical parameters

A 10-mL peripheral blood sample was drawn from each subject, after an overnight fasting for 12 h. The Biosystem A15 auto-analyzer with different enzymatic colorimetric methods was used to assess concentrations of some special biochemical parameters including: (1) triglyceride (TG) (glycerol phosphate oxidase (GPO)); (2) fasting blood glucose (FBG) (glucose oxidase (GOD)); (3) high-density lipoprotein cholesterol (HDL-c) (cholesterol oxidase (CHOD)). Other serum parameters were also measured using the commercial enzyme-linked immunosorbent assay (ELISA) kits as follows: (1) high sensitive C-reactive protein (hs-CRP) (turbidimetry kit, latex enhanced turbidimetric method, Delta.DP); (2) insulin (Monobined Inc. Lake Forest, CA 92630, USA); (3) BDNF and adropin (Zellbio, Veltlinerweg, Germany). Insulin resistance (IR) was calculated through Homeostasis Model Assessment Insulin Resistance (HOMA-IR) formula: HOMA-IR = [FBG (mmol/L) × fasting insulin (mU/L)]/22.5^37^. First decile of serum BDNF concentration was considered as low serum BDNF levels (serum BDNF < 0.47 ng/mL).

### Assessment of metabolic health status

Metabolic health of participants was evaluated using the criteria defined by Wildman et al.^38^. According to this definition, subjects with normal-weight (18.5 $\le$ BMI < 25) or overweight/obesity (BMI $\ge$ 25) were considered to have metabolically unhealthy normal-weight (MUNW) and metabolically unhealthy overweight/obese (MUOW) profiles, if they had more than two of the following risk factors: (1) high FBG levels ($\ge$ 100 mg/dL); (2) decreased HDL-c levels ($<$ 40 mg/dL for males or $<$ 50 mg/dL for females); (3) elevated TG levels ($\ge$ 150 mg/dL); (4) hypertension (BP $\ge$ 130/85 mmHg); (5) IR (HOMA-IR > 90th percentile or > 3.99); (6) elevated inflammatory protein hs-CRP levels (> 90th percentile, or > 6.14 mg/L). In contrast, other normal-weight and overweight/obese participants with less than two of the above-mentioned parameters were known as metabolically healthy normal-weight (MHNW) and metabolically healthy overweight/obese (MHOW), respectively.

### Assessment of other variables

Data on sex, age, marital status, education and smoking were collected through a self-reported questionnaire. Furthermore, a validated questionnaire was applied to evaluate socioeconomic status (SES) of participants^39^. This questionnaire assessed individuals in terms of the number of family members, homeownership, type of car, having laptops/computers and traveling in the year. Physical activity (PA) was also measured using a validated International Physical Activity Questionnaire-short form (IPAQ-SF) that evaluates three categories of activity including walking, moderate-intensity activities, and vigorous-intensity activities^40^. The data from this questionnaire were converted to Metabolic Equivalent minutes per week (MET.min/week) and participants were categorized as inactive (< 600 MET.min/week), minimally active (≥ 600 to < 3000 MET.min/week), or active (≥ 3000 MET.min/week).

### Statistical analysis

Energy-adjusted intake of legumes and nuts was computed based on residual method. Then, participants were classified according to tertiles of energy-adjusted legumes and nuts intake. Continues and categorical variables were reported as mean ± SD/SE and number (percentage), respectively. We used one-way ANOVA and chi-square test to compare characteristics of subjects across tertiles of energy-adjusted legumes and nuts intake. Dietary intakes of individuals were also assessed through ANCOVA by adjusting sex, age and energy intake. Possible associations between intake of legumes and nuts with metabolic unhealthy (MU) status and its components were determined using binary logistic regression. Confounding effects of potential variables (including age, sex and energy intake in the first model, education, marital status, smoking status, SES and PA in the second model, intake of fruits, vegetables, dairy, whole and refined grains in the third model, and BMI in the last model) were controlled in the analyses^41,42^. All odds ratios (ORs) were calculated by considering the first tertile of legumes and nuts as the reference. Tertiles of energy-adjusted legumes and nuts intake were regarded as an ordinal variable in logistic regression models to evaluate trend of ORs across tertiles of legumes and nuts intake. Stratified analyses were also conducted by BMI categories (normal-weight vs. overweight/obese) and sex (women vs. men). Multivariable-adjusted odds of low BDNF values (< 0.47 ng/mL) in tertiles of energy-adjusted legumes and nuts intake were determined by considering the effects of age, sex, PA, high BP, high TG and high FBG. In addition, linear regression analysis was applied to assess adropin levels in tertiles of energy-adjusted legumes and nuts intake with adjusting age, sex, energy intake, PA and BMI as covariates. SPSS software version 26 (IBM, Chicago, IL) was utilized for all analyses and P-values < 0.05 were regarded to be statistically significant.

### Ethical approval and consent to participate

The study procedure was performed according to declaration of Helsinki and STROBE checklist. All participants provided informed written consent. The study protocol was approved by the local Ethics Committee of Isfahan University of Medical Sciences.

### Consent to participate

Informed consent was obtained from all participants involved in the study.

## Results

This study was conducted on 527 adults (45.7% female) with an average age of 42.66 (± 11.19 SD) years. Participants had a mean weight of 75.77 (± 14.59 SD) kg and BMI range of 16.60 to 59.80 kg/m^2^. Prevalence of MU status among all participants was 42.5%, which 20.5% of them had normal weight and 79.5% had overweight/obesity.

General characteristics of participants across energy-adjusted tertiles of legumes and nuts intake are shown in Table 1. Individuals in the top tertile of legumes and nuts intake were more likely to be male, had a lower body weight and also lower prevalence of hypertriglyceridemia, compared to those at the bottom tertile. However, no substantial differences were observed in other demographic and cardiometabolic variables including age, BMI, WC, education, marital status, smoking, SES, PA, high BP, high FBG, IR (high HOMA-IR score), high hs-CRP, low HDL-c, low BDNF values and adropin levels.

**Table 1:** Demographic and cardiometabolic features of participants across energy-adjusted tertiles of legumes and nuts intake (n = 527).

Variables	Tertiles of energy-adjusted legumes and nuts intake^1^			P-value^2^
	Tertile 1 (n = 175) (< 34.26 g/day)	Tertile 2 (n = 176) (34.26–54.46 g/day)	Tertile 3 (n = 176) (> 54.46 g/day)	
Age (year)	42.36 ± 11.81	43.08 ± 10.69	42.53 ± 11.09	0.82
Body weight (kg)	78.22 ± 14.30	73.81 ± 13.27	75.31 ± 15.81	0.02
BMI (kg/m^2^)	27.17 ± 4.20	26.68 ± 4.15	26.88 ± 4.92	0.52
WC (cm)	94.04 ± 10.87	91.69 ± 11.15	92.26 ± 12.33	0.14
Sex				0.01
Male	112 (64.0)	80 (45.5)	94 (53.4)	
Female	63 (36.0)	96 (54.5)	82 (46.6)	
Education				0.28
Diploma or lower	21 (12.1)	23 (13.1)	14 (8.0)	
Higher than Diploma	153 (87.9)	152 (86.9)	160 (92.0)	
Marital status				0.71
Single	28 (16.2)	25 (14.3)	32 (18.4)	
Married	142 (82.1)	147 (84.0)	141 (81.0)	
Divorced or widow	3 (1.7)	3 (1.7)	1 (0.6)	
Smoking				0.18
Non-smoker	146 (93.6)	148 (93.1)	146 (94.2)	
Ex-smoker	2 (1.3)	8 (5.0)	5 (3.2)	
Smoker	8 (5.1)	3 (1.9)	4 (2.6)	
SES				0.79
Low	33 (30.8)	35 (31.5)	38 (32.5)	
Moderate	33 (30.8)	40 (36.0)	34 (29.1)	
High	41 (38.3)	36 (32.4)	45 (38.5)	
Physical activity levels				0.32
Inactive	99 (56.6)	100 (57.1)	98 (56.3)	
Minimally active	67 (38.3)	56 (32.0)	63 (36.2)	
Active	9 (5.1)	19 (10.9)	13 (7.5)	
High SBP (≥ 130 mmHg)	54 (30.9)	41 (23.3)	48 (27.3)	0.28
High DBP (≥ 85 mmHg)	73 (41.7)	68 (38.6)	69 (39.2)	0.82
High FBG (≥ 100 mg/dL)	39 (22.3)	37 (21.0)	28 (15.9)	0.28
High TG (≥ 150 mg/dL)	81 (46.3)	54 (30.7)	58 (33.0)	0.01
Low HDL-C (< 40 mg/dL in men, < 50 mg/dL in women)	26 (14.9)	19 (10.8)	16 (9.1)	0.22
High HOMA-IR score (> 3.99)	19 (10.9)	18 (10.2)	15 (8.5)	0.75
High hs-CRP (> 6.4 mg/L)	19 (10.9)	18 (10.2)	15 (8.5)	0.75
Low BDNF (< 0.47 ng/mL, 1st decile)	25 (14.3)	14 (8.0)	15 (8.5)	0.10
Adropin (pg/mL)	50.40 ± 21.47	59.19 ± 49.53	60.01 ± 43.12	0.06

Quantitative variables: means ± standard deviation (SD). Qualitative variables: frequency (percentage).

^1^Legumes and nuts intake was adjusted for energy intake based on residual method.

^2^Resulted from ANOVA for quantitative variables and chi-square test for categorical variables.

Dietary intakes of subjects across energy-adjusted tertiles of legumes and nuts intake are illustrated in Table 2. Participants with higher consumption of legumes and nuts had also higher intakes of proteins, dietary fiber, folate, magnesium and potassium, and lower intakes of total energy and refined grains. While, consumptions of fruits, vegetables, meats, fish, whole grains, dairy, carbohydrates, fats, vitamin C and calcium did not differ significantly across tertiles of legumes and nuts intake.

**Table 2:** Multivariable-adjusted intakes of selected food groups and nutrients of study participants across energy-adjusted tertiles of legumes and nuts intake (n = 527).

Variables	Tertiles of energy-adjusted legumes and nuts intake^1^			P-value^2^
	Tertile 1 (n = 175) (< 34.26 g/day)	Tertile 2 (n = 176) (34.26–54.46 g/day)	Tertile 3 (n = 176) (> 54.46 g/day)	
Energy (kcal/day)	2455.11 ± 50.47	2105.33 ± 50.28	2271.50 ± 49.98	< 0.001
Food groups (g/day)				
Fruits	567.72 ± 24.46	572.35 ± 24.35	528.04 ± 23.94	0.36
Vegetables	328.24 ± 17.18	358.74 ± 17.10	337.42 ± 16.81	0.45
Meats	91.66 ± 4.45	99.84 ± 4.43	104.94 ± 4.36	0.10
Fish	7.26 ± 0.71	7.82 ± 0.71	7.61 ± 0.69	0.86
Whole grains	121.41 ± 6.14	105.18 ± 6.11	111.00 ± 6.01	0.18
Refined grains	314.39 ± 12.20	244.41 ± 12.14	262.34 ± 11.94	< 0.001
Dairy	308.82 ± 20.39	338.62 ± 20.30	302.57 ± 19.95	0.41
Other nutrients				
Proteins (% of energy)	13.48 ± 0.21	14.35 ± 0.21	14.90 ± 0.21	< 0.001
Carbohydrates (% of energy)	61.78 ± 0.62	61.22 ± 0.61	59.72 ± 0.61	0.05
Fats (% of energy)	26.58 ± 0.51	26.40 ± 0.51	27.43 ± 0.50	0.31
Dietary fiber (g/day)	19.50 ± 0.48	21.00 ± 0.48	23.00 ± 0.47	< 0.001
Vitamin B9 (mcg/day)	295.13 ± 8.15	345.04 ± 8.11	385.14 ± 7.97	< 0.001
Vitamin C (mg/day)	200.18 ± 7.77	202.76 ± 7.74	192.40 ± 7.61	0.61
Magnesium (mg/day)	262.60 ± 4.89	286.55 ± 4.87	302.32 ± 4.79	< 0.001
Calcium (mg/day)	912.32 ± 28.64	953.01 ± 28.51	906.70 ± 28.03	0.46
Potassium (mg/day)	3565.38 ± 80.02	3900.98 ± 79.66	3829.54 ± 78.32	0.01

Values are means ± standard error (SE). Energy intake and macronutrients were adjusted for age and sex; all other values were adjusted for age, sex and energy intake.

^1^Legumes and nuts intake was adjusted for energy intake based on residual method.

^2^P-value obtained from ANCOVA test for adjustment of energy intake.

**Figure 1:**
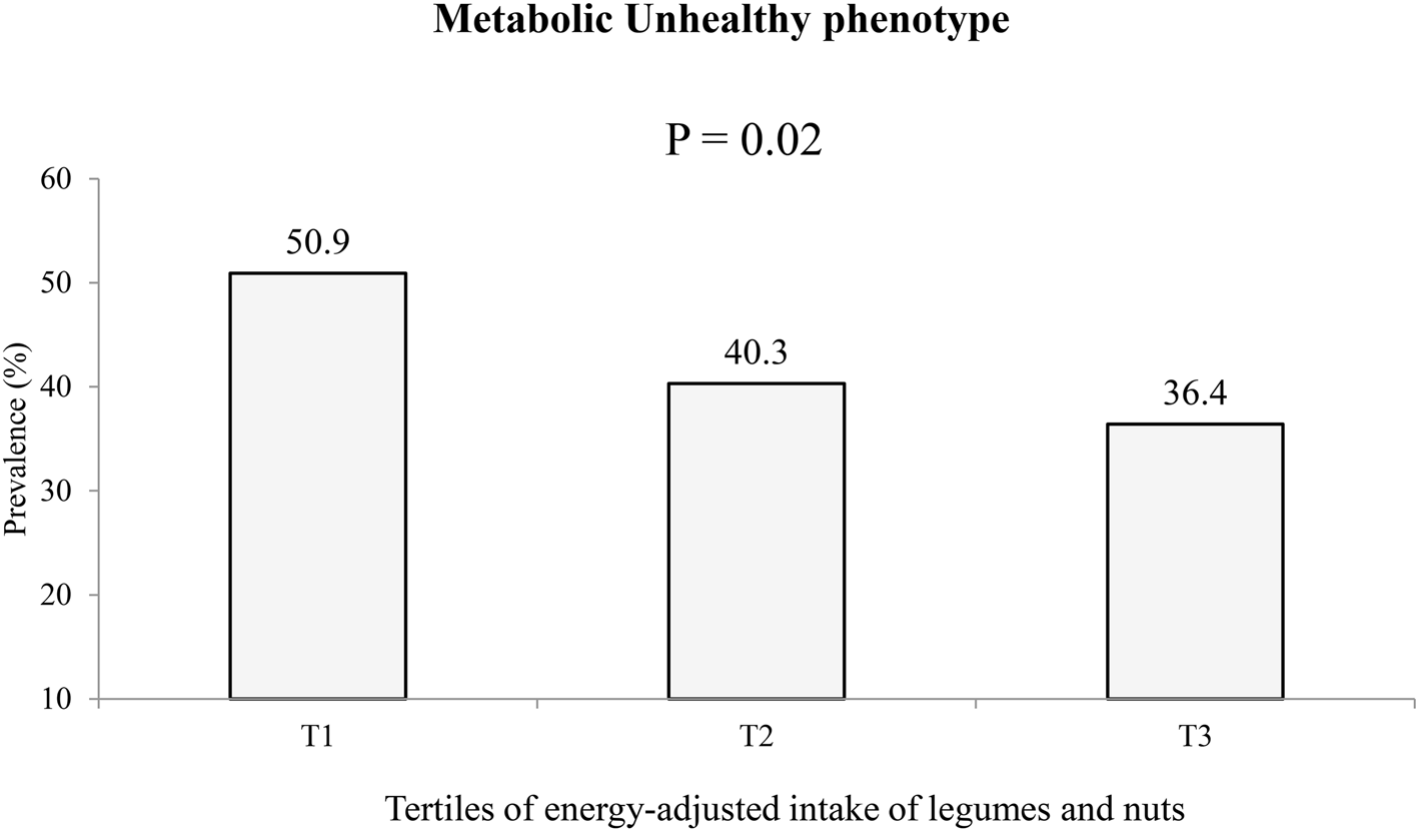
Prevalence of metabolically unhealthy (MU) status across energy-adjusted tertiles of legumes and nuts intake in the study population.

Figure 1 represents the prevalence of MU status across energy-adjusted tertiles of legumes and nuts intake. Prevalence of MU phenotype was 50.9% in first tertile, 40.3% in the second tertile and 36.4% in the third tertile of legumes and nuts intake. This prevalence was significantly different across tertiles of legumes and nuts intake (P_value_ = 0.02).

Crude and multivariable-adjusted ORs for MU phenotype across energy-adjusted tertiles of legumes and nuts intake are reported in Table 3. In comparison with the first tertile of legumes and nuts intake, individuals at the last tertile had a decreased odds of MU status in crude model (OR_T3 vs. T1_ 0.55; 95% CI 0.36–0.85). After controlling potential variables, this association was strengthened (OR_T3 vs. T1_ 0.35; 95% CI 0.18–0.71). A significant decreasing trend was also observed for MU phenotype across tertiles of legumes and nuts intake in all models (P_trend_ = 0.01). Each tertile increase in intake of legumes and nuts was significantly associated with reduced odds of MU status in crude and maximally-adjusted models as well. Stratified analysis by BMI revealed an inverse relationship between legumes and nuts intake and likelihood of MUNW profile among normal-weight participants, in both crude (OR_T3 vs. T1_ 0.35; 95% CI 0.14–0.87) and fully-adjusted (OR_T3 vs. T1_ 0.15; 95% CI 0.03–0.85) models. Among participants with overweight/obesity, legumes and nuts intake was marginally associated with reduced odds of MUOW profile in crude model (OR_T3 vs. T1_ 0.60; 95% CI 0.36–1.00). This association was strengthened after considering confounders (OR_T3 vs. T1_ 0.29; 95% CI 0.12–0.69).

**Table 3:** Multivariable-adjusted odds ratio for metabolic unhealthy status across energy-adjusted tertiles of legumes and nuts intake.

	Tertiles of energy-adjusted legumes and nuts intake^1^				Per 1 tertile increase
	Tertile 1 (< 34.26 g/day)	Tertile 2 (34.26–54.46 g/day)	Tertile 3 (> 54.46 g/day)	P_trend_	
All participants (n = 527)					
MU cases/participants (n)	89/175	71/176	64/176		
Crude	1.00	0.65 (0.43–1.00)	0.55 (0.36–0.85)	0.01	0.74 (0.60–0.92)
Model 1	1.00	0.72 (0.45–1.14)	0.56 (0.35–0.88)	0.01	0.75 (0.59–0.94)
Model 2	1.00	0.50 (0.26–0.96)	0.35 (0.18–0.67)	0.01	0.59 (0.43–0.82)
Model 3	1.00	0.51 (0.26–1.01)	0.38 (0.19–0.73)	0.01	0.62 (0.44–0.86)
Model 4	1.00	0.50 (0.25–1.00)	0.35 (0.18–0.71)	0.01	0.60 (0.42–0.85)
Normal-weight participants (n = 170)					
MU cases/participants (n)	20/57	17/57	9/56		
Crude	1.00	0.79 (0.36–1.73)	0.35 (0.14–0.87)	0.03	0.61 (0.40–0.94)
Model 1	1.00	0.75 (0.33–1.72)	0.40 (0.16–1.02)	0.06	0.64 (0.41–1.01)
Model 2	1.00	0.79 (0.26–2.37)	0.25 (0.07–0.98)	0.05	0.53 (0.28–1.01)
Model 3	1.00	0.42 (0.09–1.97)	0.15 (0.03–0.85)	0.03	0.39 (0.16–0.92)
Over-weight or obese participants (n = 357)					
MU cases/participants (n)	69/118	54/119	55/120		
Crude	1.00	0.59 (0.35–0.99)	0.60 (0.36–1.00)	0.05	0.78 (0.60–1.00)
Model 1	1.00	0.68 (0.39–1.21)	0.56 (0.32–0.98)	0.04	0.75 (0.57–0.99)
Model 2	1.00	0.36 (0.16–0.83)	0.29 (0.13–0.67)	0.01	0.55 (0.36–0.83)
Model 3	1.00	0.40 (0.17–0.95)	0.29 (0.12–0.69)	0.01	0.55 (0.36–0.84)

All values are odds ratios and 95% confidence intervals. Model 1: Adjusted for age, sex, energy intake. Model 2: More adjustments for physical activity, socioeconomic status, education, marital status, smoking status. Model 3: Further adjustments for dietary intake of fruits, vegetables, dairy, whole and refined grains. Model 4: More adjustment for BMI. P_trend_ was obtained by the use of tertiles of legumes and nuts intake as an ordinal variable in the model.

^1^Legumes and nuts intake was adjusted for total energy intake based on residual method.

Table 4 presents the association between MU status and intake of legumes and nuts, stratified by sex. Among women, an inverse association was observed between dietary intake of legumes and nuts with MU status in both crude (OR_T3 vs. T1_ 0.46; 95% CI 0.23–0.94) and fully-adjusted (OR_T3 vs. T1_ 0.28; 95% CI 0.09–0.091) models. Legumes and nuts intake of men was not substantially related to MU phenotype in crude model (OR_T3 vs. T1_ 0.68; 95% CI 0.40–1.19). However, by adjusting potential confounders this association became marginally significant (OR_T3 vs. T1_ 0.40; 95% CI 0.16–1.00).

**Table 4:** Multivariable-adjusted odds ratio for metabolic unhealthy status across energy-adjusted tertiles of legumes and nuts intake stratified by sex.

	Tertiles of energy-adjusted legumes and nuts intake^1^				Per 1 tertile increase
	Tertile 1 (< 34.26 g/day)	Tertile 2 (34.26–54.46 g/day)	Tertile 3 (> 54.46 g/day)	P_trend_	
Women (n = 241)					
MU cases/participants (n)	26/63	31/96	20/82		
Crude	1.00	0.68 (0.35–1.31)	0.46 (0.23–0.94)	0.03	0.68 (0.48–0.97)
Model 1	1.00	0.77 (0.38–1.58)	0.56 (0.26–1.17)	0.12	0.75 (0.51–1.08)
Model 2	1.00	0.30 (0.10–0.95)	0.24 (0.08–0.72)	0.01	0.49 (0.28–0.87)
Model 3	1.00	0.31 (0.10–0.98)	0.25 (0.08–0.78)	0.02	0.51 (0.29–0.90)
Model 4	1.00	0.30 (0.09–0.97)	0.28 (0.09–0.91)	0.04	0.53 (0.29–0.96)
Men (n = 286)					
MU cases/participants (n)	63/112	40/80	44/94		
Crude	1.00	0.78 (0.44–1.38)	0.68 (0.40–1.19)	0.17	0.83 (0.63–1.09)
Model 1	1.00	0.70 (0.38–1.29)	0.57 (0.32–1.03)	0.06	0.76 (0.56–1.01)
Model 2	1.00	0.67 (0.29–1.53)	0.44 (0.19–1.00)	0.05	0.66 (0.44–1.00)
Model 3	1.00	0.71 (0.28–1.82)	0.51 (0.22–1.22)	0.13	0.72 (0.47–1.10)
Model 4	1.00	0.69 (0.26–1.86)	0.40 (0.16–1.00)	0.05	0.63 (0.40–1.00)

All values are odds ratios and 95% confidence intervals. Model 1: Adjusted for age, and energy intake. Model 2: More adjustments for physical activity, socioeconomic status, education, marital status, smoking status. Model 3: Further adjustments for dietary intake of fruits, vegetables, dairy, whole and refined grains. Model 4: More adjustment for BMI. P_trend_ was obtained by the use of tertiles of legumes and nuts intake as an ordinal variable in the model.

^1^Legumes and nuts intake was adjusted for total energy intake based on residual method.

Crude and multivariable-adjusted ORs for metabolic health components across energy-adjusted tertiles of legumes and nuts intake are shown in Table 5. In crude model, participants with the highest intake of legumes and nuts had lower odds of hypertriglyceridemia and hypertension, compared to the lowest intake. However, by controlling all confounders, higher consumption of legumes and nuts was significantly associated with decreased likelihood of hyperglycemia (OR_T3 vs. T1_ 0.38; 95% CI 0.16–0.89), hypertriglyceridemia (OR_T3 vs. T1_ 0.48; 95% CI 0.25–0.92), hypertension (OR_T3 vs. T1_ 0.37; 95% CI 0.18–0.76), and marginally related to reduced odds of low HDL-cholesterolemia (OR_T3 vs. T1_ 0.41; 95% CI 0.16–1.09) and high hs-CRP levels (OR_T3 vs. T1_ 0.39; 95% CI 0.14–1.07).

**Table 5:** Multivariable-adjusted odds ratio for metabolic components across tertiles of legumes and nuts intake.

	Tertiles of energy-adjusted legumes and nuts intake^1^			P_trend_
	Tertile 1 (n = 175) (< 34.26)	Tertile 2 (n = 176) (34.26–54.46)	Tertile 3 (n = 176) (54.46 >)	
Hyperglycemia (FBG ≥ 100 mg/dL)				
Crude	1.00	0.93 (0.56–1.54)	0.66 (0.39–1.13)	0.13
Multivariable-adjusted^2^	1.00	0.82 (0.37–1.82)	0.38 (0.16–0.89)	0.02
Hypertriglyceridemia (TG ≥ 150 mg/dL)				
Crude	1.00	0.51 (0.33–0.80)	0.57 (0.37–0.88)	0.01
Multivariable-adjusted^2^	1.00	0.54 (0.27–1.06)	0.48 (0.25–0.92)	0.03
Low HDL-cholesterolemia^3^				
Crude	1.00	0.69 (0.37–1.31)	0.57 (0.30–1.11)	0.09
Multivariable-adjusted^2^	1.00	0.35 (0.13–0.97)	0.41 (0.16–1.09)	0.08
Hypertension (BP ≥ 130/85 mmHg)				
Crude	1.00	0.72 (0.47–1.09)	0.64 (0.42–0.98)	0.04
Multivariable-adjusted^2^	1.00	0.50 (0.24–1.03)	0.37 (0.18–0.76)	0.01
Insulin resistance (HOMA-IR score ≥ 3.99)				
Crude	1.00	0.94 (0.47–1.85)	0.77 (0.38–1.56)	0.46
Multivariable-adjusted^2^	1.00	0.83 (0.30–2.31)	0.87 (0.31–2.43)	0.80
High hs-CRP (> 6.4 mg/L)				
Crude	1.00	0.94 (0.47–1.85)	0.77 (0.38–1.56)	0.46
Multivariable-adjusted^2^	1.00	0.43 (0.15–1.23)	0.39 (0.14–1.07)	0.08

All values are odds ratios and 95% confidence intervals. P_trend_ was obtained by the use of tertiles of legumes and nuts intake as an ordinal variable in the model.

*FBG* fasting blood glucose, *TG* triglycerides, *HDL* high density lipoprotein, *BP* blood pressure, *HOMA-IR* homeostasis model assessment insulin resistance, *hs-CRP* high sensitive C-reactive protein.

^1^Legumes and nuts intake was adjusted for total energy intake based on residual method.

^2^Adjusted for age, sex, energy intake, physical activity, socioeconomic status, education, marital status, smoking status, dietary intake of fruits, vegetables, dairy, whole and refined grains and BMI.

^3^HDL-c < 40 mg/dL in men, and < 50 mg/dL in women.

**Figure 2:**
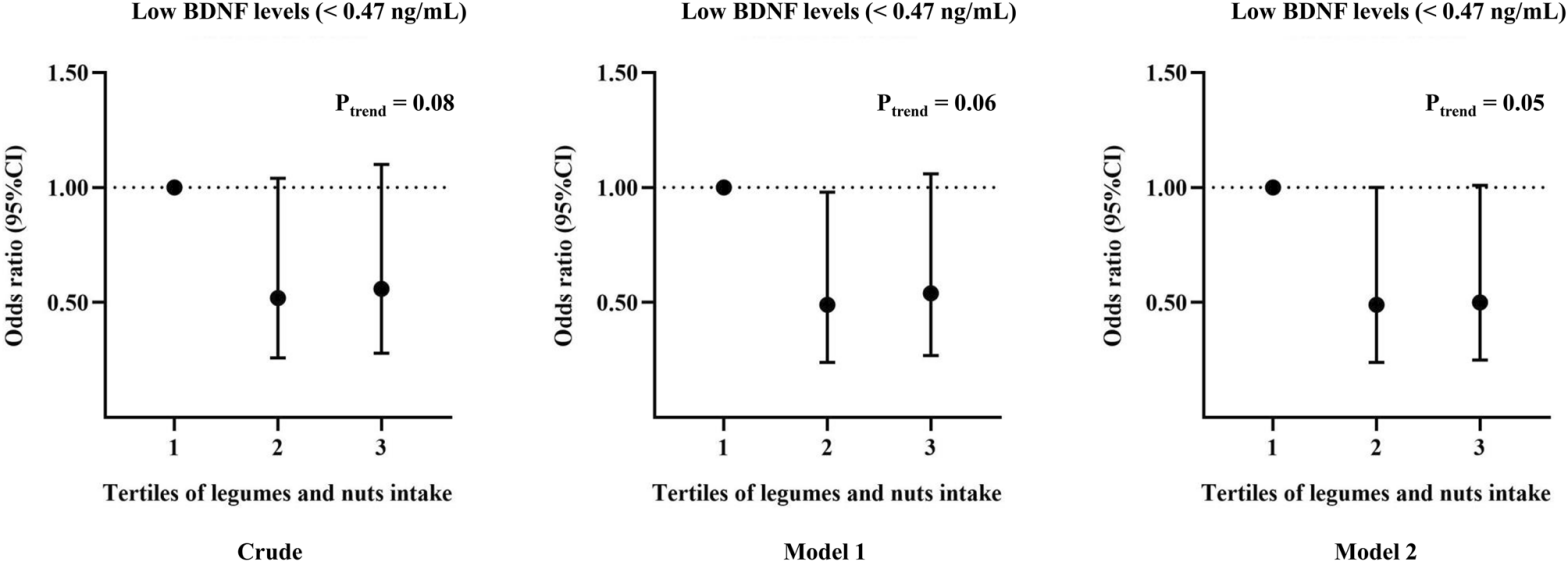
Multivariable-adjusted odds ratio and 95% confidence intervals for low BDNF levels across tertiles of legumes and nuts intake. Model 1: Adjusted for age and sex; Model 2: More adjustments for physical activity, history of high blood pressure, high triglyceride and high fasting blood glucose. P_trend_ was obtained by the use of tertiles of legumes and nuts intake as an ordinal variable in the model.

**Figure 3:**
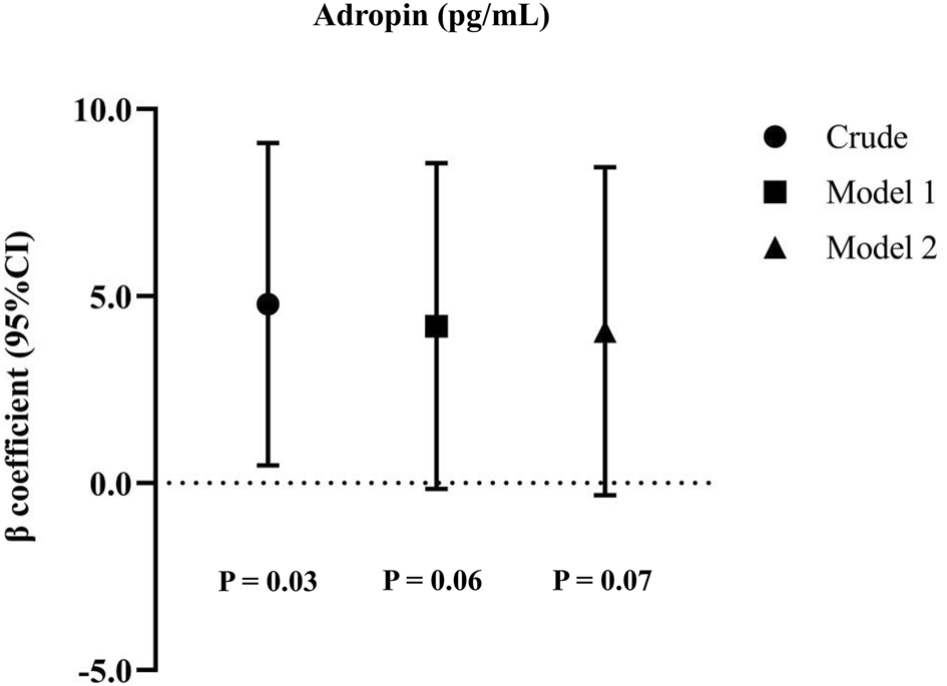
Linear association between tertiles of legumes and nuts intake with adropin levels. All values are regression coefficients and 95% confidence intervals. Tertiles of legumes and nuts intake were considered as an ordinal variable in linear regression analysis. Model 1: Adjusted for age, sex, and energy intake; Model 2: More adjustments for physical activity and BMI.

The average serum levels of BDNF and adropin among participants were 1.25 ng/mL and 56.59 pg/mL, respectively. Multivariate-adjusted ORs for low BDNF levels across tertiles of legumes and nuts intake are depicted in Fig. 2. Compared to the reference group, higher intake of legumes and nuts was marginally related to decreased likelihood of low BDNF levels, in both crude (OR_T3 vs. T1_ 0.56; 95% CI 0.28–1.10) and multivariable-adjusted (OR_T3 vs. T1_ 0.50; 95% CI 0.25–1.01) models. As shown in Fig. 3, linear regression analysis revealed that each tertile increment in legumes and nuts intake was substantially associated with an increase of 4.78 pg/mL in levels of adropin in crude model (95% CI 0.47–9.09; P_value_ = 0.03). After adjusting all covariates, a marginally significant direct association was observed between each tertile increase in legumes and nuts intake with adropin levels ($\beta$ = 4.06; 95% CI − 0.32, 8.44; P_value_ = 0.07). No significant differences were found in circulating BDNF and adropin levels between individuals with metabolically healthy and unhealthy phenotypes, even after stratifying analysis by sex or age categories (Supplemental Figs. 1, 2).

## Discussion

The findings of the present cross-sectional study showed that more than 40% of the Iranian population had an MU phenotype with a significantly higher prevalence among obese/overweight than normal-weight individuals (79.5% vs. 20.5%). Higher consumption of legumes and nuts was associated with lower odds of MU phenotype both in normal-weight and obese/overweight individuals. This association was more prominent among females. A significant inverse relationship was also found between legumes and nuts intake with some components of metabolic health status including hyperglycemia, hypertriglyceridemia, and hypertension. These beneficial associations might be facilitated through higher BDNF and adropin levels.

Non-communicable diseases (NCDs), mainly CVDs, cancers, respiratory diseases, and diabetes, account for over 70% of deaths worldwide^43^. Among the modifiable lifestyle behaviors increasing the risk of NCDs, diet plays a key role through its contribution in metabolic disturbances^44^. Therefore, dietary intake assessments and implementing early clinical interventions could be beneficial approaches for reducing risk of metabolic disturbances and related NCDs. Findings of this study revealed that daily consumption of more than 54 g of legumes and nuts could reduce MU odds. Therefore, providing nutritional education and interventions to consume a healthy diet containing appropriate amounts of legumes and nuts is recommended as a beneficial approach to reduce risk of MU status.

To the best of our knowledge, this report is the first observational study investigating the relationship between the intake of legumes and nuts with metabolic health status. However, legume and nut intake in relation to MetS or its components has been investigated previously and revealed controversial results. Contrary to our results, a cross-sectional study involving 420 Iranian female nurses has failed to find any association between the consumption of legumes and nuts with MetS or its components^28^. Additionally, a systematic review and meta-analysis of 7 observational studies containing 56,028 participants demonstrated no significant association between legume intake and odds of MetS^41^. However, the beneficial effect of legume intake on total cholesterol and low-density lipoprotein cholesterol (LDL-c) as well as BP in obese and overweight individuals has been reported by two other studies^45,46^. A meta-analysis of 11 observational studies showed an inverse significant association between nut intake and risk of MetS^42^. There are also other reports on the protective role of nut consumption on cardiometabolic disorders. Nut intake has been shown to have a favorable effect on HOMA-IR and fasting insulin in a meta-analysis of 40 randomized controlled trials^47^. In addition, the beneficial effect of nut consumption on hypertension has been reported in other studies^48^. However, in a meta-analysis of 6 randomized controlled trials, no significant association was found between total nut intake and lipid profile^49^. These discrepant findings can be explained by different genetic backgrounds and dietary habits of the studied population, tools used to assess dietary intakes, and controlling for various confounders.

Many NCDs in adulthood, especially endocrine dysfunctions, have roots in health status, lifestyle and dietary intakes in childhood. According to a cohort survey by Liang et al., children with obesity had higher risk of diabetes in adulthood^50^. Another study also revealed that childhood risk factors including unhealthy diets had long-term effects on metabolic health and CVD risk in adulthood^51^. Therefore, maintaining the health status in early life would be of great importance in every society. In our previous study, we discovered that higher intake of legumes and nuts was associated with lower odds of metabolic unhealthy among Iranian adolescents aged 12 to 18 years^33^. The same findings were obtained in the current investigation in adults. Although there were no sufficient data regarding the metabolic status of the present study participants in their childhood, it could be mentioned that the current metabolic status of these individuals might be affected by their childhood lifestyle. Therefore, it would be more valuable to modify food habits and intakes from childhood and adolescence in order to prevent the occurrence of many diseases in adulthood.

Our results showed that the inverse association between legumes and nuts intake with MU phenotype was more prominent in females. This finding might be explained by healthier dietary knowledge and behavior among women than men^52^. Also, better metabolic profiles among women due to the differences in substrate use, accumulation and metabolism in important metabolic organs including liver, skeletal muscle and fat tissue may explain the observed finding^53^. Furthermore, premenopausal women have reduced odds of metabolic disturbances which highlights the key role of sex-hormones in metabolic health^53^. In addition, the observed association was stronger among normal-weight rather than over weight/obese subjects. Different physiological responses to environmental factors, such as diet, and differences in accuracy of reported food intakes might explain the observed findings. However, further studies are required to confirm these hypotheses.

We found a marginally inverse association between low BDNF levels with legumes and nuts intake. A positive linear association was also observed between levels of adropin and legumes and nuts intake. So far, several studies have examined the association between some dietary factors with BDNF and adropin levels in both animals and humans^12,13,16,54^. A clinical trial conducted on Spanish adults found that the Mediterranean diet supplemented by nuts was related to reduced odds of low BDNF levels^55^. There were no other related investigations that examined the association of legumes or nuts intake with BDNF and adropin levels. The current epidemiologic study revealed no substantial differences in BDNF or adropin levels between metabolically healthy and unhealthy groups. Although some previous studies have documented that higher concentrations of BDNF and adropin were related to decreased odds of metabolic disorders through regulating energy hemostasis and its related signaling pathways^7–9^, there are insufficient data regarding this association and the possible mechanisms. Our findings could broaden insights into further related studies.

The inverse relationship between the intake of legumes and nuts with MU phenotype can be explained by several feasible mechanisms. Legumes and nuts are rich in fiber, minerals, and bioactive compounds which might have beneficial effects on metabolic health status. Magnesium content of legumes and nuts plays an important role in decreasing inflammation and insulin resistance^56,57^. Additionally, a large body of evidence indicates that dietary fiber contributes in improving metabolic health status components such as hypertension, dyslipidemia, insulin sensitivity, and inflammatory biomarkers levels^58^. Another explanation for the beneficial influence on metabolic health status may be the antioxidant and anti-inflammatory properties of bioactive phytochemicals such as polyphenols which have modulatory effects on metabolic processes^59^. Moreover, low glycemic index of legumes and nuts reduces the risk of insulin resistance and improves metabolic health status^60^.

The current study has several limitations that should be acknowledged. The study does not provide evidence for a causal relationship between the consumption of legumes and nuts with MU, due to its cross-sectional design. Self-reported dietary intakes might be subject to recall bias and misclassification, despite using a validated FFQ for assessment of dietary intakes. In addition, due to low amounts of nuts intake in our study population, no separate analysis was performed for nuts and legumes intake. On the other hand, to the best of our knowledge, this is the first study that investigates the links between the intake of legumes and nuts with metabolic health status in a somehow representative sample of Iranian adults. Furthermore, a relatively comprehensive definition of metabolic health status (Wildman et al. method) was used that includes an inflammatory index (hs-CRP) in addition to HOMA-IR and traditional cardiometabolic risk factors. The confounding role of several variables was also taken into account in the statistical analyses. The final strength of the study was the assessment of serum levels of adropin and BDNF which rarely have been investigated in epidemiological studies of nutrition.

In summary, a significant relationship was found between consumption of legumes and nuts with decreased odds of MUNW and MUOW phenotypes, particularly among females. Higher consumption of legumes and nuts was also inversely associated with hyperglycemia, hypertriglyceridemia, and hypertension. The association might be facilitated through BDNF and adropin. Further population-based prospective studies are warranted to confirm these findings.

### Supplementary Information

Supplementary Figures.

## Data Availability

The data that support the findings of this study are available from the corresponding author upon reasonable request.

## Abbreviations

BP: Blood pressure
BMI: Body mass index
BDNF: Brain-derived neurotrophic factor
CI: Confidence interval
CHOD: Cholesterol oxidase
CVDs: Cardiovascular diseases
DASH: Dietary approach to stop hypertension
DBP: Diastolic blood pressure
ELISA: Enzyme-linked immunosorbent assay
FBG: Fasting blood glucose
FFQ: Food frequency questionnaire
GOD: Glucose oxidase
GPO: Glycerol phosphate oxidase
HDL-c: High-density lipoprotein cholesterol
HOMA-IR: Homeostasis model assessment insulin resistance
hs-CRP: High sensitive C-reactive protein
IR: Insulin resistance
IPAQ-SF: International physical activity questionnaire-short form
LDL-c: Low-density lipoprotein cholesterol
MET: Metabolic equivalent
MetS: Metabolic syndrome
MHNW: Metabolically healthy normal-weight
MHO: Metabolically healthy obesity
MHOW: Metabolically healthy overweight/obesity
MU: Metabolically unhealthy
MUNW: Metabolically unhealthy normal-weight
MUO: Metabolically unhealthy obesity
MUOW: Metabolically unhealthy overweight/obesity
NCDs: Non-communicable diseases
OR: Odds ratio
PA: Physical activity
SBP: Systolic blood pressure
SD: Standard deviation
SE: Standard error
SES: Socioeconomic status
T: Tertile
TG: Triglycerides
WC: Waist circumference
