# Supplementary material for: Legumes and nuts intake in relation to metabolic health status, serum brain derived neurotrophic factor and adropin levels in adults
Source: Sci Rep. 2023 Sep 30;13:16455. doi: 10.1038/s41598-023-43855-8 (PMC10542386; doi:10.1038/s41598-023-43855-8)
Supplement: Supplementary file 1 — Supplementary Figures. [file 41598_2023_43855_MOESM1_ESM.docx]

**Legumes and nuts intake in relation to metabolic health status, serum brain derived neurotrophic factor and adropin levels in adults**

Mohammad Javad Assi et al.

**"Online Supplementary Material"**


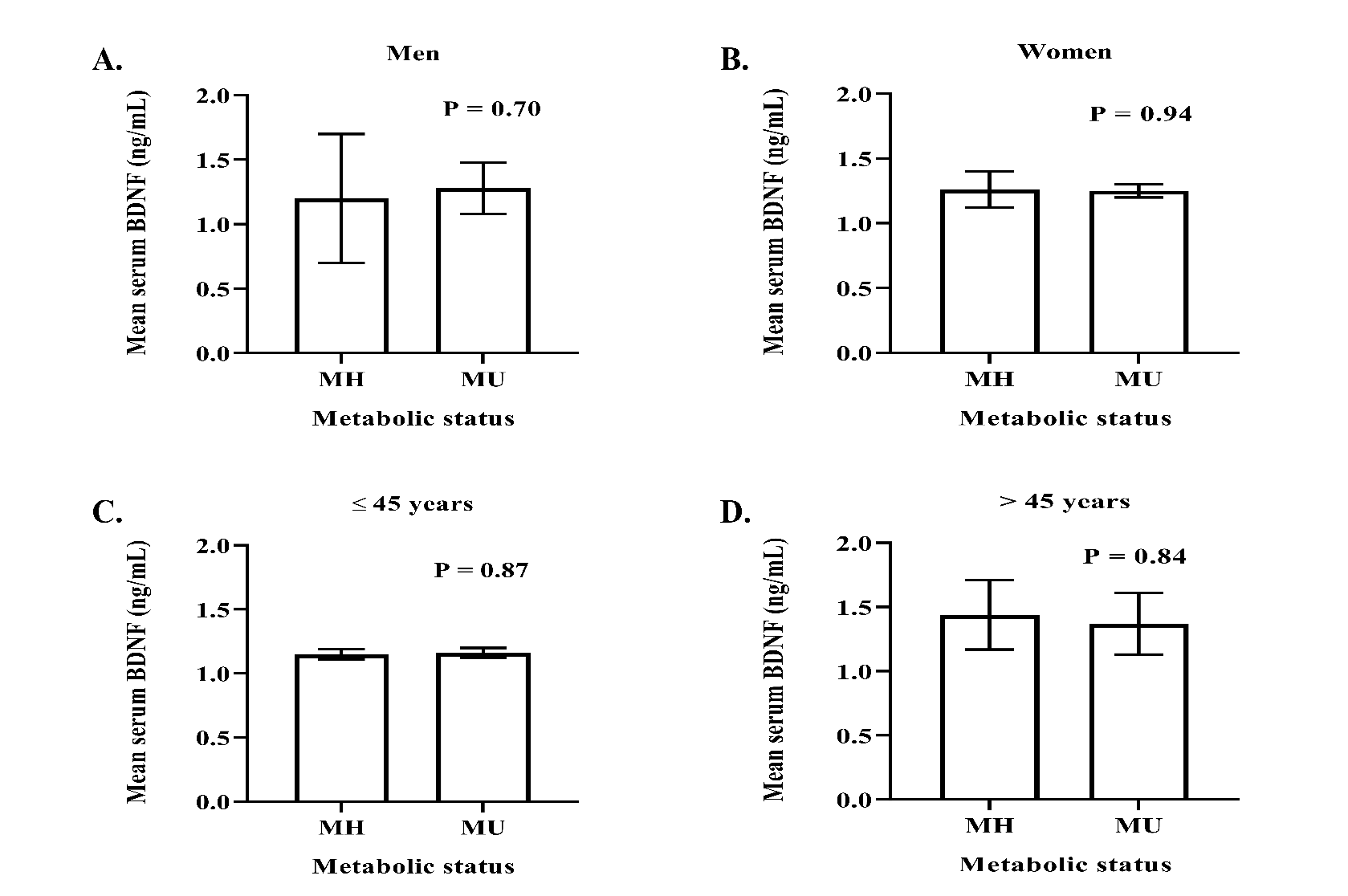


**Supplemental Figure 1.** Mean (± standard error) serum BDNF levels across metabolic status categories (metabolically healthy (MH) vs. metabolically unhealthy (MU)), stratified by sex (**A.** men, **B.** women) and age categories (**C.** $\leq$ 45 years, **D.** > 45 years).


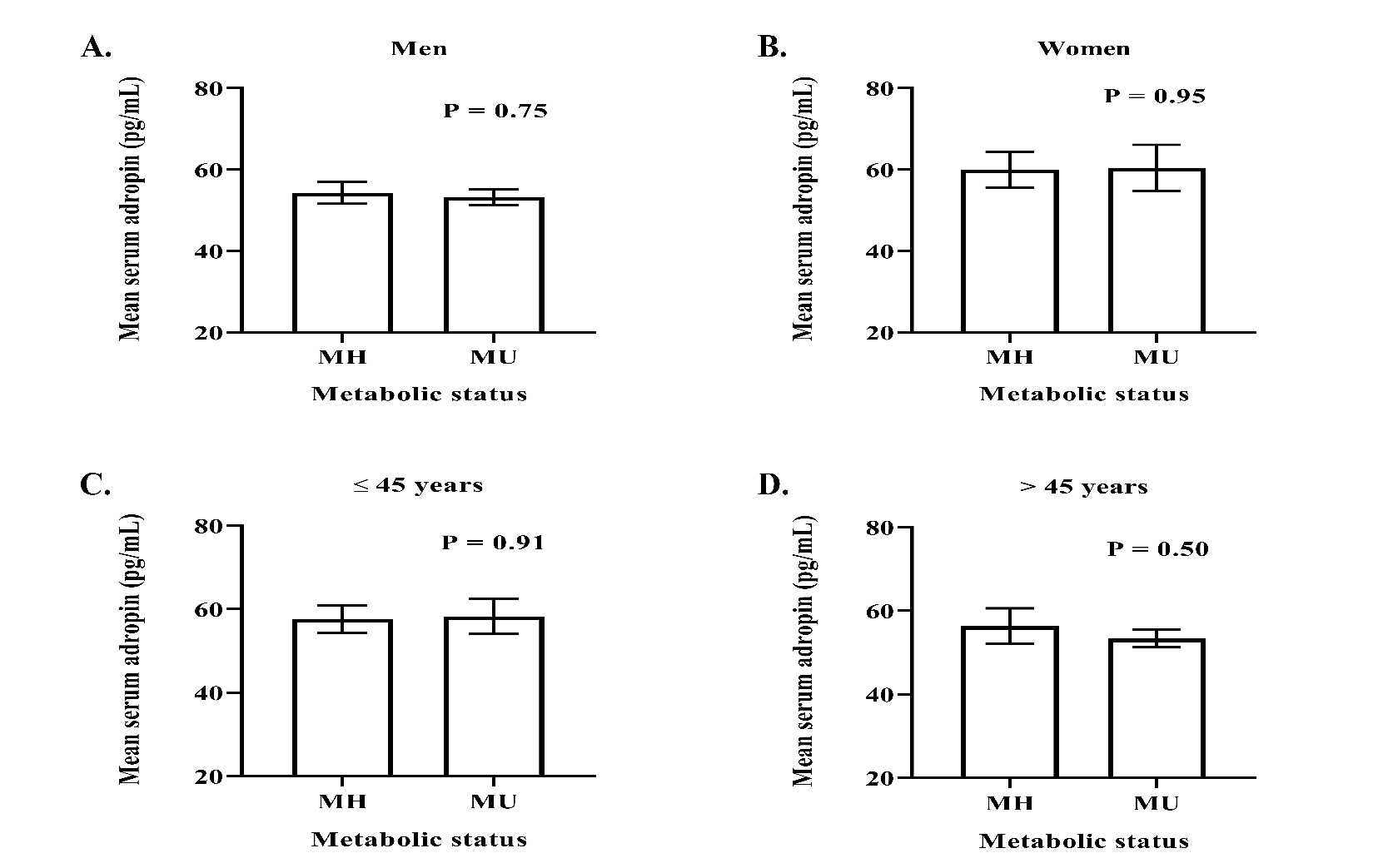


**Supplemental Figure 2.** Mean (± standard error) serum adropin levels across metabolic status categories (metabolically healthy (MH) vs. metabolically unhealthy (MU)), stratified by sex (**A.** men, **B.** women) and age categories (**C.** $\leq$ 45 years, **D.** > 45 years).
